# Senescence in Human Mesenchymal Stem Cells: Functional Changes and Implications in Stem Cell-Based Therapy

**DOI:** 10.3390/ijms17071164

**Published:** 2016-07-19

**Authors:** Valentina Turinetto, Emanuela Vitale, Claudia Giachino

**Affiliations:** Department of Clinical and Biological Sciences, University of Turin, 10043 Orbassano, Turin, Italy; emyx-90@libero.it (E.V.); claudia.giachino@unito.it (C.G.)

**Keywords:** mesenchymal stem cells, senescence, differentiation potential, immunoregulatory activity, migratory ability, tumour-promoting function

## Abstract

Regenerative medicine is extensively interested in developing cell therapies using mesenchymal stem cells (MSCs), with applications to several aging-associated diseases. For successful therapies, a substantial number of cells are needed, requiring extensive ex vivo cell expansion. However, MSC proliferation is limited and it is quite likely that long-term culture evokes continuous changes in MSCs. Therefore, a substantial proportion of cells may undergo senescence. In the present review, we will first present the phenotypic characterization of senescent human MSCs (hMSCs) and their possible consequent functional alterations. The accumulation of oxidative stress and dysregulation of key differentiation regulatory factors determine decreased differentiation potential of senescent hMSCs. Senescent hMSCs also show a marked impairment in their migratory and homing ability. Finally, many factors present in the secretome of senescent hMSCs are able to exacerbate the inflammatory response at a systemic level, decreasing the immune modulation activity of hMSCs and promoting either proliferation or migration of cancer cells. Considering the deleterious effects that these changes could evoke, it would appear of primary importance to monitor the occurrence of senescent phenotype in clinically expanded hMSCs and to evaluate possible ways to prevent in vitro MSC senescence. An updated critical presentation of the possible strategies for in vitro senescence monitoring and prevention constitutes the second part of this review. Understanding the mechanisms that drive toward hMSC growth arrest and evaluating how to counteract these for preserving a functional stem cell pool is of fundamental importance for the development of efficient cell-based therapeutic approaches.

## 1. Introduction

The maintenance and repair of adult tissues and organ are guaranteed by the adult stem cell pool. Among adult stem cells, mesenchymal stem cells (MSCs) are emerging as hopeful candidates for cell-based therapy of numerous diseases.

Human MSCs (hMSCs) are non-hematopoietic cells capable of self-renewal and multi-lineage differentiation into various tissues of mesodermal origin. These cells can be easily isolated and expanded from the stroma of virtually all organs, although the preferred sources are bone marrow and subcutaneous fat [1]. Upon isolation, hMSCs are characterized by their capability to adhere to plastic, develop as fibroblast colony-forming-units, and differentiate into osteocytes, chondrocytes, and adipocytes. After in vitro culture expansion, hMSCs are positive for CD73, CD90, and CD105 and negative for CD11b, CD14, CD34, CD45, and HLA-DR [2]. In addition to their robust differentiation potential, hMSCs have a remarkable capacity of inhibiting the immune response, an activity known generally as immunomodulation or immunoregulation [3]. This hMSC immunoregulatory capacity includes B and T cell proliferation inhibition, cytokine production inhibition, together with decreased NK cell activation, and dendritic cell maturation. By taking advantage of these features, hMSCs have been used successfully in several experimental models for allogeneic transplant rejection prevention and autoimmune/inflammatory disorders treatment. Notably, most of the beneficial effects mediated by hMSC cell therapy involved the broad repertoire of secreted trophic factors (commonly referred to as the MSC secretome) exhibiting diverse functions such as immunomodulation, anti-inflammatory activity, angiogenesis regulation, and anti-apoptotic activity. Due to their high proliferative potential, multipotency, paracrine effect, and immunomodulatory activity, MSCs are ideal candidates for regenerative medicine and immunotherapy [4,5]. Indeed, MSCs are the major stem cells for cell therapy and in the last decade have been used in the clinic to treat a variety of traumatic and degenerative disorders [6].

Cultured primary cells do not grown infinitely, but undergo only a limited number of cell division, in a process called cellular senescence [7], and hMSCs make no exception. Although hMSCs are present in several tissues, they are scarce in the body. For this reason cell therapy protocols generally require hundreds of million hMSCs per treatment and, consequently, these cells need to be expanded in vitro for about 10 weeks before implantation (http://www.clinicaltrials.gov). Notably, patient’s clinical history, age, and genetic makeup strongly influence the length of this expansion period and the quality of the obtained cells [8,9]. Aged MSCs generally perform less well than their younger counterparts in various disease models (reviewed in [10]) and mounting evidence strongly suggests that cellular senescence contribute to aging and age-related diseases. In particular, the factors that senescent cells secrete affect vital and tightly-regulated processes, such as cell growth and migration, tissue architecture, blood vessel formation, and differentiation. The inappropriate presence of these factors can disrupt tissue structure and function. Among the secreted factors, there are also several potent inflammatory cytokines. Chronic inflammation is a hallmark of aging that initiates or supports most major age-related diseases. Chronic inflammation and the strong oxidants produced by some immune cells can affect cell and tissue quality tissues because some immune cells produce strong oxidants. In addition, other factors secreted by the immune cells further alter and remodel the tissue enviroment, promoting cell/tissue dysfunction and stem cell niche impairment. Oxidatine damage and the general inflammatory milieu can also initiate carcinogenesis and promote cancer by suppressing immune surveillance and stimulating malignant phenotypes (reviewed in [11,12,13]).

Due to the prolonged expansion regimens that are needed in the clinic to obtain sufficient amounts of hMSCs for therapy, and based on the patient-specific quality of cells, it is quite likely that long-term culture evokes continuous changes in hMSCs. In particular, a substantial proportion of cells may undergo senescence [14]. It would, thus, be of great significance to monitor the occurrence of a senescent phenotype in hMSCs addressed to clinical uses and to evaluate the functional consequences of senescence in hMSCs which could affect their clinical therapeutic potential, taking into account their paracrine effects, immunomodulatory activity, differentiation potential, and cell migration ability [14].

## 2. Senescence Activation in hMSCs and Phenotypic Characterization

Both exogenous and endogenous factors constantly stress and damage cells, including stem cells. Additionally, to either complete recovery and cell survival or cell death, proliferating cells can undertake a third response by adopting a state of permanent cell-cycle arrest, termed cellular senescence.

Cellular senescence was formally described in the 1960s when Hayflick and colleagues showed that human diploid fibroblasts had a limited ability to proliferate in culture [7]. Subsequently, it was demonstrated that mitotically-competent cells respond to many other stressors by undergoing cellular senescence. These stressors include dysfunctional telomeres, genotoxic stresses/DNA damage, perturbations to chromatin organization, and strong mitogenic signals. Notably, activation of the DNA damage response (DDR) pathways is involved in both the induction and maintenance of senescence in many cases. In this way, cellular senescence can be regarded as a permanent DNA damage response activation. Shortening telomeres produces a persistent DDR, which activates and sustains the senescence growth arrest [15,16,17,18,19]. DNA double-strand breaks are specifically potent senescence activators [20]. In addition, compounds which relax chromatin without substantially damaging DNA, such as histone deacetylase inhibitors, activate the DDR proteins Ataxia Telangiectasia Mutated (ATM) and the p53 tumour suppressor [21], and induce a senescence response [22,23]. Finally, strong mitogenic signals, such as those delivered by certain oncogenes or highly-expressed pro-proliferative genes can induce cells to senescence [24,25,26]. In this case misfired replication origins and replication fork collapse are causative of DNA damage and a persistent DDR. [27,28,29]. Thus, many senescence-inducing stimuli cause epigenomic interference or genomic damage.

At the molecular level, senescence is triggered by the retinoblastoma protein (Rb) or p53 pathways, which activate the cyclin-dependent kinase inhibitors p16 and p21, respectively. Notably, the pathways can act on each other and cooperate to induce senescence [30,31,32].

Senescent cells undergo irreversible growth arrest but continue to be metabolically active and develop a large, flat morphology, display characteristic changes in gene expression, typically exhibit a senescence-associated β-galactosidase (SA-β-gal) activity, harbour characteristic enlarged and persistent DNA damage nuclear foci (PDDF) that contain DDR proteins, including γH2AX and 53BP1, and accumulate a distinct heterochromatin structure, termed senescence-associated heterochromatin foci (SAHFs) [19]. In addition, senescent cells secrete a myriad of factors, including growth factors, proteases and cytokines, with potent autocrine and paracrine activities [33,34,35,36,37]. Some of the biological features of senescent cells can be explained by this senescence-associated secretory phenotype (SASP).

Human MSCs have been reported to be highly resistant to apoptosis induced by different genotoxic insults [38,39,40,41] and preferentially respond to injury with activation of stress-induced premature senescence (SIPS). Wang et al. published the first comprehensive phenotypic (and mechanistic) study of X-ray induced senescence in hMSCs [42]. Ten days after irradiation, the majority of the cells became enlarged, flattened, and stained positive with SA-β-gal. Analysing expression profiles of Rb, p53, p21, and p16, they confirmed IR-induced hMSC senescence, highlighting that the critical transition occurred between days 3 and 6. In particular, their results suggested that p53-p21 pathway played a key role in IR-induced cell cycle arrest as shown previously [43], whereas the the Rb-p16 pathway might play a more important role in IR-induced full senescence of hMSCs. Additionally, they partially described the hMSC SASP phenotype, reporting significant upregulation of Growth Related Oncogene (GRO), IL8, IL12, and Macrophage-Derived Chemokine (MDC). Finally, they characterized the cytoskeletal reorganization of hMSC, describing a reduction of myosin-10, redistribution of myosin-9 and secretion of profilin-1 and demonstrating that Ck2 was a crucial kinase involved in this reorganization [42].

**Figure 1:**
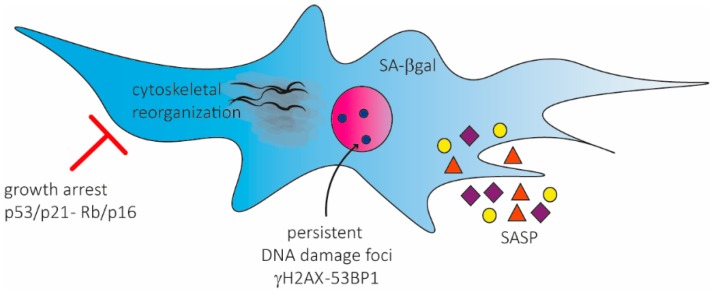
Phenotypic characterization of senescent hMSCs. Senescent hMSCs activate p53/p21 and Rb/p16 pathways to block the cell cycle and sustain growth arrest. Senescent hMSCs are characterized by a specific SASP and by the presence of persistent DNA damage foci, containing γH2AX and 53BP1, and are positive for SA-β-gal.

Senescence activation in hMSCs was described in some other papers, using hMSCs from different sources, such as dental pulp [44,45,46], cord blood [47], and endometrium [48,49]. Human MSCs respond with a senescence program following different stresses, including oxidative stress [49,50], heat shock [48], and chemotherapeutic agents [46,51,52]. These works demonstrated that the senescence activation pathway and resulting profile are independent of the tissue source and the stress stimuli. They also added some details in hMSC senescence characterization. In particular, analogously to human fibroblasts [18], hMSC senescence was sustained by persistent DDR activation, as highlighted by the presence of characteristic enlarged (PDDF), containing γH2AX and 53BP1 foci [44,46,51] (Figure 1).

Additionally, Sepulveda et al. characterized in details the SASP activated in senescent hMSCs and measured global gene expression changes following senescence induction [53]. Senescent hMSCs secreted higher levels of numerous proteins compared to non-senescent cells: they identified 27 proteins that were over-secreted by senescent cells in comparison to non-senescent cells. Among the factors that reached the highest levels of secretions, they reported LEPTIN, Transforming Growth Factor Alpha (TGFA), IL8, EOTAXIN, Interferon Gamma (IFNG), VCAM1, Interferon Beta (IFNB), IL4, and Monocyte Chemotactic Protein-1 (MCP1). Notably these factors are relevant for their ability to exacerbate the inflammatory response at a systemic level (Figure 1). In accordance with previous reports [54,55], genome expression profile analysis detailed more than 5000 genes, including 31 miRNAs, differentially expressed in senescent hMSCs compared to control cells. These genes affect several cellular functions, including cell growth and proliferation, cell cycle, cell death, and cellular movement. Considering miRNA expression profile, it was reported an upregulation of the miR-34 family, which had been previously demonstrated to to be regulated by p53 and to induce cell senescence [56,57]. In contrast, the miR-17 group was downregulated in senescent cells, analogously to the decreased expression observed in human aging [58].

In addition to SIPS, replicative senescence has been widely studied in hMSCs, particularly for its clinical implications. Once in culture, hMSCs undergo replication senescence, by some estimates between 50 and 90 days post-harvest [59]. Mechanisms and molecular pathways leading to hMSC replicative senescence are analogous to those involved in SIPS. p16, p21, and p53 are among the senescence-related genes upregulated in long-term culture [60]. The tumour suppressor Rb genes have also been shown to play a role in hMSC senescence [61]. Specific changes in gene expression have been observed during long-term MSC culture. Generally, these changes included downregulation of genes related to differentiation, focal adhesion organization, cytoskeletal maintenance, and mitochondrial function [52].

## 3. Functional Alterations in Senescent hMSCs

Many reports investigated the effect of hMSC senescence on migratory ability, differentiation potential, immunomodulation ability, and tumor progression.

**Figure 2:**
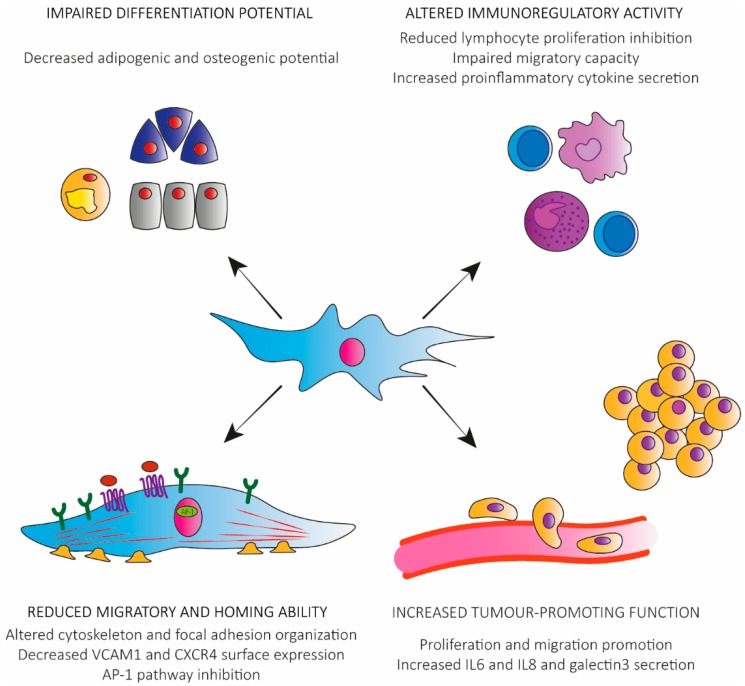
Functional alterations occurring in senescent hMSCs. Senescent hMSCs exhibit impaired differentiation potential, altered immunoregulatory activity, reduced migratory, and homing ability, and increased tumour-promoting functions.

With senescence, hMSCs exhibited decreased differentiation potential and the balance between differentiations to the osteogenic versus adipogenic lineages was disrupted, although the direction of this shift is still controversial. Some studies indicated that the osteogenic activity of hMSCs deteriorated progressively as a function of increasing lifespan [62]. On the other hand, there were numerous reports in which the osteogenic potential in late passage MSCs was preserved or even increased [54,63,64]. Accumulation of oxidative stress and dysregulation of key differentiation regulatory factors such as Runx2, C/EBPα, and PPARγ [60,65] appeared to be crucial in this balance loss. Disagreement about whether senescent MSCs are more or less osteogenic is likely an effect of differing culture conditions and the lack of optimal in vitro assays to fully characterize osteogenesis. For example, increased cell death can determine a higher degree of alizarin red staining, providing the false suggestion of enhanced osteogenic differentiation. In one well-controlled study employing a rat model, long-term cultured MSCs revealed a great in vitro differentiation potential impairment, with complete loss of osteogenic potential and diminished adipogenic potential [55] (Figure 2).

Recently, Sepulveda et al. addressed how cellular senescence influences the therapeutic potential of hMSCs, testing their immunoregulatory activity in vitro and in vivo [53]. Data demonstrated that radiation-induced senescence abrogated the hMSC protective immunoregulatory function effect in a mouse model of sepsis. On one hand, senescent hMSCs retained an aptitude to regulate the inflammatory response on macrophages in vitro and, in part, retained their ability to inhibit lymphocyte proliferation, but on the other hand they had a severely reduced migratory capacity in response to proinflammatory signals, which was associated with an inhibition of the AP-1 pathway. Notably, many of the SASP components over-secreted by senescent hMSCs are associated to the immune system process. For example, IL8 induces chemotaxis in neutrophils and other granulocytes [66], and is a potent promoter of angiogenesis [67]. VCAM1 mediates leukocyte-endothelium adhesion, and elevated levels of circulating soluble form has been related to systemic inflammation disease, such as systemic lupus erythematosus and coronary artery disease [68,69]. Finally, MCP1 (CCL2) is a chemoattractant for monocytes and basophils and has an important role in several inflammatory diseases, such as multiple sclerosis [70] and inflammatory bowel disease [71]. These data strongly support the idea that in vivo administration of senescent hMSCs could exacerbate the inflammatory response at a systemic level and counteract the anti-inflammatory effect of these cells as measured in vitro. Understanding the physiological and pathological factors that influence the immune modulatory activity of hMSCs is of great importance, for both autoimmune/inflammatory disorders and degenerative pathologies. Indeed, recent data clearly demonstrated that the regenerative capabilities of transplanted MSCs in damaged tissues, such as infarcted myocardium, mainly reside in their paracrine activity, instead of their potential for differentiation into specific cell lineages [72,73] (Figure 2).

For an effective therapeutic approach based on MSCs, a proper cell migration towards relevant stimuli is crucial for functional engraftment. In the previously described paper by Sepulveda et al. [53], a migratory defect in senescent hMSCs was associated with their altered immunomodulation ability. The authors demonstrated a close association of AP-1 pathway inhibition with the impaired response of senescent hMSCs to migratory stimuli. Measuring the expression of the AP-1 components FOS and JUN (and their phosphorylated forms) after application of a migratory stimuli (macrophage-conditioned medium), they found that senescent hMSCs showed a marked decrease in the expression of these proteins. To further confirm the relation between defective AP-1 activation and migratory impairment, they evaluated FOS expression in an in vitro wound-healing assay, and observed that the majority of presenescent wound-edge cells showed a high FOS expression soon after the scraping of the cell monolayer, whereas senescent wound-edge hMSCs exhibited weak FOS levels at the same time point [53]. In a subsequent paper, authors correlated miR-355 increase in senescent hMSCs with AP-1 activity inhibition [74]. Many other papers reported that MSCs lost their migratory and homing ability during in vitro expansion culture, albeit most of these data referred to murine models [55,75,76,77]. Rombouts et al. showed that primary bone marrow derived MSCs were able to efficiently home and expand in both spleen and bone marrow in an irradiated syngeneic mouse model. Differently, in vitro-expanded MSCs rapidly lost their in vivo homing ability [77]. Geissler et al. assessed in vitro migration potential of rat MSCs after long-term cultivation and described a significantly decreased migration rate of in vitro aged MSCs compared to their primary counterparts [55]. A transcriptome analysis revealed that genes involved in focal adhesion organization and cytoskeleton turnover were downregulated in long-term cultured MSCs [55]. Notably, cellular migration strongly depends on local cytoskeleton organization and actin turn-over [78] and the impact of local actin organization for migration is accented by the importance of lamellipodia, fillopodia and focal complex formation for cellular migration [79]. Declining maintenance of the cytoskeleton and focal adhesion machinery was observed with serial MSC passaging [80]. On the molecular level, a downregulation of mRNAs for several chemokines, cytokines, and their receptors relevant for cell migration was observed during in vitro aging, e.g., stromal cell-derived factor 1 (SDF-1) and its receptor Chemokine Receptor Type 4 (CXCR4) [55]. Studies investigating skeletal repair and systemic skeletal disorders in animal models showed that CXCR4 and SDF-1 recruited MSCs to the fracture site and prevented bone loss [81]. Changing in the MSC surface markers during prolonged cultivation was reported to be related with decreased homing ability of hMSCs [76]. In particular, the authors described a strong decrease in VCAM1 expression, an important mediator of MSC interaction with endothelial cells and subsequent MSC homing (Figure 2).

Despite senescence-induced growth arrest being a potent cell-autonomous tumour suppressor mechanism, the associated SASP, altering the behaviour of neighbouring cells and the quality of tissue environments, exerts cell-non-autonomous effects than can be either beneficial or detrimental [82]. The SASP has been shown to promote the proliferation of pre-malignant and malignant epithelium [83], enhance invasion [84], induce an epithelial to mesenchymal transition in carcinoma cells [85], increase the growth of xenograft tumors in vivo [86], and mediate paracrine transmission of senescence [87]. Nevertheless, all of these studies were conducted on senescent fibroblasts. On the other hand, it is well documented that MSCs can also play tumour-promoting functions [88,89,90]. Additional effects of senescence on MSC tumour-promoting functions were investigated and the available data highlighted the ability of senescent hMSCs to further promote either proliferation or migration (or both) of cancer cells [51,52,91,92]. Skolekova et al. demonstrated that senescent hMSCs, secreting high amount of IL6 and IL8, were able to increase the resistance of breast cancer cells to cisplatin, favoured the appearance of cancer stem-like cells in vitro, and increased cisplatin resistance and tumour volume in vivo [52]. The importance of IL6 as a principal mediator for the tumour-promoting activity of senescent hMSCs was also evidenced by Di et al., who demonstrated that its secretion stimulated the proliferation and migration of breast cancer cells in vitro and, notably, did so in vivo in a co-transplant xenograft mouse model [92]. In accordance with these data, we found recently a robust increase of several inflammatory cytokine genes in senescent hMSCs [51], including IL6, IL8, the chemokine GRO1, MCP-2 and RANTES, the inflammatory factor Granulocyte-Macrophage Colony-Stimulating Factor (GM-CSF), the metalloprotease MMP3 and the adhesion molecule Intercellular Adhesion Molecule 1 (ICAM). These senescent hMSCs, by altering their secretory profile, enhanced the in vitro migration of two solid tumour-derived cell lines [51]. In another work, galectin-3 was among the factors secreted by senescent adipose-derived MSCs that could promote tumourigenesis [91]. Galectin-3 is a member of a family of β-galactoside binding proteins and has emerged as an important regulator of diverse functions critical in cancer biology, supporting chemoresistance and metastasis in solid tumours, as well as in leukaemia and lymphoma [93] (Figure 2).

Overall, these data suggest that SASP increases the complexity of paracrine communication among hMSCs and their physiological/pathological microenvironment, further enhancing their tumour-promoting behaviour. A greater understanding of the molecular mechanisms involved in the senescence of hMSCs and careful investigation of the interconnection among senescent and naïve hMSC secretory phenotypes will provide valuable new insights to comprehend the link with this cancer-promoting behaviour.

## 4. Tools to Monitor hMSC Senescence in Vitro

There is a growing perception that standardized protocols and quality control of therapeutic cell preparations are a prerequisite for reliable and reproducible cellular therapy and that these should include cellular senescence assessment. Different methods have been tested to track hMSC senescence, with the specific aim to identify potential quantitative markers for pre-evaluating the clinical efficacy of individual hMSC preparations in clinical application (Table 1).

At a first glance, one of the most convenient predictive indicators of replicative senescence in vitro would appear to be the number of cell passages. However, as there is great variation both in seeding densities and time of harvesting [94,95,96,97], passage numbers may lead to deceptive results under non-standardized conditions. To evaluate the number of cumulative population doublings (PD) should overcome these limitations [98]. Yet, analysis of PD does not consider the probable events of cells lost for apoptosis, necrosis or during passaging. More importantly, there are large variations between different donor samples. Taken together, these data suggest it to be hard to predict at which passage or number of cell divisions MSCs are approaching a senescent state.

A popular hystochemical approach is to stain the senescent cells based on the accumulation of SA-β-gal. The staining procedure is easy and reliable but the results can hardly be quantified and almost exclusively the enlarged, late senescent cells stain positive for SA-β-gal [54,99], precluding detection of the early senescent cells. In addition, SA-β-gal is not required for manifestation of senescence. Despite these limitations, SA-β-gal staining is presently the most widely used biomarker for senescent cells.

Telomere length decrease may serve as another possible indicator for mitotic history and the prospective additional life span [100,116]. However, stress induced senescence may occur independently of cell division and telomere shortening and it needs to be demonstrated if quantitative analysis of telomere length facilitates reliable and prospective quality control with regard to cellular aging.

A still debated theme concerns the genetic stability of in vitro expanded hMSCs and how it could drive hMSC towards a senescence phenotype. It has been shown that hMSCs do not transform spontaneously in vitro and chromosomal instability and cytogenetic aberrations occur without leading to malignant transformation [101,102]. It is becoming more accepted that all ex vivo hMSC expansion procedures favour the accumulation of aneuploid cells, which is intimately associated with the progression of senescence [101,103,104,117]. Anyway, the observed cytogenetic aberrations prompted to evaluate conventional karyotyping and CGH array (complete genome hybridization-array) approaches as a further ways to monitor hMSCs senescence [105,106]. However, through conventional karyotyping minor genomic gains or losses may not be detected, while the more sensitive CGH array technique is incapable of revealing balanced translocations or very small mutations. More importantly, there are presently no clearly-described karyotype markers for cellular aging.

Considering the limitations highlighted for these previously described methods, based on either phenotypic or cytogenetic changes that occur in senescent hMSCs, more recently novel efforts were turned towards evaluation of either gene expression or DNA methylation changes during long-term cultures.

Replicative senescence-associated gene expression changes in hMSCs, isolated in two different laboratories and grown under different culture conditions, were analysed with different microarray platforms [107]. Despite these methodological differences, there was a high resemblance in senescence-associated gene expression signatures. Authors found senescence-associated upregulation of PTPL1-associated RhoGAP 1 (PARG1) and cyclin-dependent kinase inhibitor 2B (CDKN2B) and down-regulation of pleiotrophin (PTN) mini-chromosome maintenance complex component 3 (MCM3) [107]. To validate these result, they performed quantitative RT-PCR analysis of these four genes in other five different types of MSC preparations and long-term cultures from different laboratories [108]. Despite confirming the described differences between early and late passages, standard deviations were rather high, impeding distinguishing senescent MSCs clearly.

In a more recent paper, hMSCs derived from six different donors, grown under identical culture conditions and harvested at cell passages 3, 5, and 7, were analysed with gene-expression profiling by using microarray technology [109]. Their analysis revealed a set of 78 genes that significantly differ in expression between the first and the last passage analysed. When the significant gene lists were analysed through pathway analysis, almost 40% of these genes turned out to be involved cellular growth, proliferation, and cellular development. Notably, this gene expression profile of senescent MSC suggests that extensively cultured MSCs seem to be undergoing differentiation. These results identified specific gene markers to distinguish in vitro aged MSCs [108].

Other similar investigations involved gene-expression profiling of human MSCs derived from bone marrow upon long-term culture. Kulterer et al. reported that 838 genes were differentially expressed between P2 and P5, with as few as 10 genes matching those identified in Ballayr study (*BST1*, *COL11A1*, *COL12A1*, *GALNT5*, *HAS1*, *KRT18*, *MEG3*, *PCM1*, *PENK*, and *SHB*) [110]. Likewise, in another study by Tanabe et al., only two reported genes matched those found in Ballayr paper (*KRT18* and *PRDX4*) [111]. A number of reasons exist for these discrepancies from a biologic standpoint, including culture conditions, media used, and the source of MSCs.

Further specification of senescence-associated markers and cross-validation in different MSC preparations are needed for a reliable quality control of cell preparations based on gene expression level. Additional work is necessary to determine whether the expression of identified gene markers is consistently modulated when cellular expansion is scaled up by using bioreactors or performed with different substrates and media components. Discovering genes expressed in MSCs that correlate with a functional outcome will provide a basis for a set of quality markers. These quality markers can then be used to assess cellular products desired for a specific application. These are major considerations in establishing quality, function, and safety of MSCs for therapeutic purposes.

Additionally, through whole genome expression profiling, it was demonstrated that DNA methylation profiles are clearly affected by long-term culture [112]. Culture expansion of MSCs is associated with senescence-associated DNA-methylation (SA-DNAm) changes at specific promoter regions, which become either hyper-methylated or hypo-methylated [105,112,113]. These reports evidenced that in long-term culture hypermethylation increases at genes related to DNA replication, cell cycle regulation, DNA repair, adipogenic differentiation, and several metabolic processes, such as genes for lipid and fatty acid metabolic process. Importantly these data highlighted that SA-DNAm changes are highly reproducible and may therefore be used to monitor cellular senescence. To this end, Wagner group elaborated an Epigenetic-Senescence-Signature based on six specific CpG sites, which seemed to display consistent SA-DNAm changes in different cell preparations. Two CpG sites, associated with *GRM7* and *CASR* genes, become continuously hyper-methylated in long-term culture and four CpG sites, associated with *PRAMEF2*, *SELP*, *CASP14*, and *KRTAP13-3* genes, become hypo-methylated. Integration of these DNAm levels in linear-regression models facilitated prediction of passage number, cumulative PD, and days of in vitro culture [114]. They further validated this method on cell preparations isolated under good manufacturing practice (GMP) conditions, using cells isolated in serial passages and with DNA directly extracted from cryopreserved samples [115]. The authors demonstrated that the epigenetic senescence signature reflected inter-individual differences and variation in subpopulations, which are not necessarily mirrored in conventional long-term growth curves [115]. In this regard, the cell epigenetic state might even provide the more accurate measurement for cellular aging.

In conclusion, though to date there are no single effective methods to monitor in vitro hMSC senescence and all proposed approaches present with some limitation, the evaluation of either gene expression or DNA methylation profiles have recently provided powerful perspectives. Further bioinformatic analyses of datasets and validation enrolling different MSC preparations will hopefully pave the way for a reliable panel of distinct aging and senescence markers.

## 5. Tools to Prevent in Vitro hMSC Senescence

Some researchers have reported in vitro treatments that could improve hMSC performance. Genetic engineering of cells is one possible approach for preventing in vitro aging. Some groups have attempted to combat replicative senescence or improve MSC potency by induced ectopic expression of telomerase [118,119]. However, this approach is inadvisable for clinical applications given the possible risk of malignant transformation and/or induced tendency toward osteogenesis [120,121,122]. Another strategy relied on RB silencing. In cells with silenced RB2, it was reported DNA damage, apoptosis, and senescence reduction, along with proliferation rate and clonogenic ability, increase. Cells with silenced RB2 were cultivated for extended periods without any signs of transformation; however, silencing of RB genes disrupts differentiation to osteogenic, chondrogenic, and adipogenic lineages [61].

Oxidative stress is one of the major insults accelerating cell senescence in vivo, as well as in vitro [123]. Reduction of oxidative stress, by lowering oxygen tension or adding anti-oxidants, such as vitamin C or *N*-acetylcysteine, has been shown to prolong replicative lifespan of human cells, including hMSCs in vitro [124,125,126,127]. Isothiocyanates, reducing oxidative stress and protecting hMSCs from chemically induced oxidative damage, may contribute to slowing the aging process related to oxidative DNA damage [128]. Addition of 3% hydrogen gas to expansion cultures has been shown to extend the replicative lifespan of MSCs while maintaining intact differentiation potential [129]. Notably, hydrogen gas treatment did not diminish paracrine activity of hMSCs, but slightly altered the paracrine profiles. For example, hydrogen gas treatment increased both bFGF and HGF secretions whereas it decreased Vascular Endothelial Growth Factor (VEGF) secretion [129].

Pharmacological approaches may also be employed as tools to prevent hMSC senescence in culture. For example, decreased expression of histone deacetylases was observed in senescent MSCs; consequently, the use of a histone acetyltransferase inhibitor prevented replicative senescence of MSCs [130]. Additionally, lysophosphatidic acid (LPA), critical for membrane phospholipid synthesis, has been shown to play a role in hMSC senescence, and pharmacologic antagonism of the LPA receptor pathway achieved an anti-aging effect in cultured hMSCs, resulting in extended rounds of cellular proliferation, increased clonogenic potential, and retained plasticity for osteogenic and adipogenic differentiation [131].

It has been reported that inhibition of the phosphatidylinositol 3-kinase Akt/mTOR pathways could provide an environment to maintain MSCs in their immature undifferentiated state during long-term culture expansion. Human MSCs cultured in the presence of rapamycin showed morphology of cells in their early passages, retained their clonogenic ability, and showed a high proliferative rate and osteogenic potential. It was further demonstrated that loss of Akt/mTOR may mediate these effects by regulating the production of cytoplasmic ROS, expression of pluripotency genes *Nanog* and *Oct-4*, and by reducing accumulation of DNA damage during aging of MSCs [132]. Additionally, it has been demonstrated that rapamycin is also able influence the MSC senescent inflammatory phenotype [133]. Authors showed that bone marrow-derived-MSCs from systemic lupus erythematosus (SLE) patients exhibited senescent behaviour and were involved in the pathogenesis of SLE. Rapamycin treatment was able to reverse the senescent phenotype and improved immunoregulation. After transwell culture of CD4+ T cells with MSCs, the ratio of Treg/Th17 generated in the presence of the rapamycin-treated SLE MSCs was increased compared to those cultured in the presence of the untreated SLE MSCs. Results showed that rapamycin-treatment induced the secretion of IL-10 and TGF-β, two critical differentiation factors for the generation of Treg cells [134]. On the other side, rapamycin-treatment downregulated IL-17 and IL-6, the main factors involved in pro-inflammatory Th17 cell development [135]. Thus, their data demonstrated that rapamycin improves the immunoregulatory capacity of MSCs from SLE patients and indicated the involvement of the mTOR signalling pathway in the immune disorders of SLE patients [132].

The attempt to maintain hMSC self-renewal and differentiation potential through selected growth factors and medium supplements leave limited success. In particular, medium supplementation with fibroblast growth factor (FGF)-2, platelet-derived growth factor (PDGF)-BB, ascorbic acid (AA), and epidermal growth factor (EGF) both increased proliferation rate and markedly increased number of cell doublings before reaching senescence, with a greater than 1000-fold increase in total cell numbers for AA, FGF-2, and PDGF-BB, compared with control cultures. However, long-term culture was associated with loss of osteogenic/adipocytic differentiation potential, particularly with FGF-2 supplementation [136,137].

Finally, many studies have reported successful derivation of functional MSCs from induced pluripotent stem cells (iPSCs), referred to as induced MSCs (iMSCs) [138].The iMSCs are transpiring as an attractive source of MSCs because, during reprogramming process, cells undergo rejuvination, exhibiting better cellular vitality, such as survival, proliferation, and differentiation potentials [138]. The iMSCs could be easily scaled up to more than 40 passages while stably maintaining normal diploid karyotype, and consistent gene expression and surface antigen profile [139]. Analogously, Wei et al. demonstrated that human iMSCs could continuously proliferate for more than 32 passages without undergoing cellular senescence and displayed superior wound healing and pro-angiogenic properties [140]. Notably, the iMSCs retained donor-derived DNA methylation (DNAm) profile. However, it is important to highlight that while tissue-specific and age-related DNAm profiles of iMSCs were completely erased, the iMSCs reacquired SA-DNAm [141]. The same study also contrastingly underlines that iMSCs reacquire incomplete immunomodulatory functions [141]. Eventually, the safety of both viral and non-viral reprogramming strategies has to be taken in account for considering iPSCs and the derived iMSCs as suitable candidates for biotherapeutics. Several challenges thus need to be effectively tackled before iMSCs could be favourably used for translational applications.

Much further research is required to determine if such approaches could be safe and effective on MSC-based cell therapy. Improving the conditions of ex vivo culture and discovering better markers of MSC aging may help to suppress and monitor in vitro aging.

## Figures and Tables

**Table 1:** Methods to monitor changes in senescent hMSCs.

Method	Advantages	Limits	REF.
Phenotypic changes evaluation			
Number of passage	Simple and easily documented.	Seeding density and confluence degree variations between laboratories	[94,95,96,97]
	Good indicator for long-term culture under standardized conditions		
Cumulative population doublings	Robust parameter for comparison between different laboratories	Big variations between different samples	[98]
SA-β-galactosidase	Almost specific senescent marker. Fast and easy method	Difficult quantitative analysis. Inability to detect early senescent cells	[54,99]
Telomere length	Direct measure for prospective analysis of potential cell division.	Stress-induced senescence might be independent of telomere shortening	[100]
	Availability of several techniques to quantify telomere length		
Cytogenetic techniques			
Karyotype	Tumorigenic mutations and potentially immortalized cell clones may be detected	Minor genomic losses or gains may not be detected	[101,102,103,104]
CGH array	More sensitive technique	Inability to reveal balanced translocation or very small mutations	[105,106]
Genomic and epigenomic analyses			
Gene expression markers	Fast and reliable quantification based on microarray techniques.	Cross-validation enrolling different MSC preparation is needed	[107,108,109,110,111]
	Panels of up- and down-regulated genes may be more robust than individual markers		
DNA methylation	Senescence-associated DNA-methylation changes are highly reproducible.	Cross-validation enrolling different MSC preparations is needed	[112,113,114,115]
	Identification of an Epigenetic-Senescence-Signature based on six specific CpG sites to estimate the state of cellular aging		
